# Deep Brain Stimulation: Eye Movements Reveal Anomalous Effects of Electrode Placement and Stimulation

**DOI:** 10.1371/journal.pone.0032830

**Published:** 2012-03-12

**Authors:** Chrystalina A. Antoniades, Philip Buttery, James J. FitzGerald, Roger A. Barker, Roger H. S. Carpenter, Colin Watts

**Affiliations:** 1 Nuffield Department of Clinical Neurosciences, University of Oxford, Oxford, United Kingdom; 2 Department of Neurology, Addenbrooke's Hospital, Cambridge, United Kingdom; 3 Department of Physiology, Development and Neuroscience, University of Cambridge, Cambridge, United Kingdom; 4 Department of Neurosurgery, Addenbrookes Hospital, Cambridge, United Kingdom; 5 Cambridge Centre for Brain Repair, University of Cambridge, Cambridge, United Kingdom; Tokyo Medical and Dental University, Japan

## Abstract

One of the major difficulties in evaluating the efficacy of deep brain stimulation (DBS), or understanding its mechanism, is the need to distinguish the effects of stimulation itself from those of the lesion inevitably created during surgery. Recent work has shown that DBS of the subthalamic nucleus in Parkinson's disease greatly reduces the time it takes the eyes to make a saccade in response to a visual stimulus. Since this saccadic latency can be rapidly and objectively measured, we used it to compare the effects of surgery and of stimulation. We used a saccadometer to measure the saccadic latencies of 9 DBS patients (1) preoperatively, (2) the day after insertion of subthalamic nucleus electrodes, (3) three weeks later, prior to turning on the stimulator, and (4) after commencement of stimulation. Patients were on their anti-Parkinsonian medication throughout the study. It revealed an entirely unexpected and puzzling finding. As in previous studies an amelioration of symptoms is seen immediately after surgery, and then a further improvement when finally the stimulator is turned on, but in the case of saccadic latency the pattern is different: surgery produces a transient increase in latency, returning to baseline within three weeks, while subsequent stimulation reduced latency. Thus the differential effects of electrode placement and stimulation are completely different for saccades and for more general motor symptoms. This important finding rules out some over-simple interpretations of the mechanism of DBS, and needs to be taken into account in future attempts at modelling the neurophysiology of DBS.

## Introduction

High frequency stimulation of the subthalamic nucleus (STN) is a surgical procedure indicated for the treatment of advanced Parkinson's disease (PD) that has become resistant to other interventions and medication. It is effective for bradykinesia, rigidity, on-off effects, and tremor [1], [2], [3] and by virtue of dose reduction can ameliorate levodopa-induced dyskinesias [4], [5]. Its effects are long lasting [6], [7], [8]. Apart from its therapeutic benefits in alleviating the symptoms of PD, deep brain stimulation (DBS) of the STN has also been shown previously to produce consistent, large and robust reductions in saccadic latency [9], [10], [11], [12], i.e. the time taken to initiate an eye movement to look at a novel visual target [10], [11]. Since with modern miniaturised, non-invasive equipment, several hundred individual measurements of saccadic latency (saccadometry) can be made in a matter of minutes, this can potentially provide a more objective and qualitative way of evaluating the effects of DBS. Further benefits of saccadometry are that it provides an internationally-standardized task, and also that we have a widely accepted and applied model, LATER, that enables one to relate the findings to the presumed underlying neural decision mechanisms (see for instance [13], [14], [15], [16]).

The mechanisms that underlie the effects of deep brain stimulation (DBS) in PD remain mysterious: it has been argued by some that it increases neural activity, by others that it decreases activity, and by still others that it produces more complex effects such as interference with pathological rhythms [12], [17], [18], [19], [20], [21], [22]. An example of this complexity is the common experience that there is an improvement in Parkinsonian symptoms immediately after insertion of stimulator leads, despite the stimulator not yet being active [23], [24], in other words that the *operation itself* causes amelioration. This is a transient phenomenon and disappears over a period of days to a few weeks, to be replaced by a more sustained therapeutic effect when the stimulator is switched on [1], [2], [7], [25], [26], [27].

Saccadometry therefore seemed to us a good way of trying to compare changes in behaviour due to the stimulation itself from those due to the lesion that is inevitably created by the insertion of the stimulator leads at the time of surgery, more precisely and objectively than conventional clinical evaluation, which is necessarily imprecise and subjective. As we report, what we found was unexpected and more than a little puzzling: that the huge effects of electrode placement and electrical stimulation on latency do not correspond in direction with the normal sequence of clinical amelioration. Whereas from a clinical point of view both the surgery of electrode placement, and the actual turning on of the current both reduce the clinical manifestations of the condition, surgery makes saccadic latency worse, but the eventual stimulation makes it much better. This surprising finding rules out some over-simple interpretations of how DBS works.

## Materials and Methods

### Patients

A total of nine patients underwent bilateral STN DBS, seven male and two female, mean age 64.4 years (range 38–73). Mean disease duration prior to DBS was 9.4 years (range 4–14). The indication for surgery was either severe motor fluctuations with dyskinesias (6 patients) or treatment resistant tremor (3 patients). Of the patients with treatment resistant tremor, one also had motor fluctuations and another had medication intolerance. See table 1 for more details. This study was approved by the Cambridge Research Ethics Committee and was conducted at Addenbrooke's Hospital, Cambridge, UK. All participants gave their written informed consent after the procedures had been explained to them.

**Table 1:** Clinical details of patients undergoing DBS.

Demographics						Preoperative							24 hrs postop(OFF stim)	3 weeks postop(ON stim)		
Patient	Age	Sex	Yrs fromdiagnosis	Side ofonset†	Indicationfor surgery	UPDRS III	Tremor*ON/OFF	Rigidity*ON/OFF	Bradykinesia*ON/OFF	UPDRS IV	HY	LED	UPDRS III	UPDRS III	UPDRS IV	HY
1	73	M	5	R	3	44	6/9	5/9	13/20	2	3	687	33	27	1	3
2	70	F	8	L	1	38	4/10	5/9	10/17	10	3	801	35	30	0	2
3	38	M	4	L	2	34	11/11	3/7	4/7	12	3	600	30	27	2	3
4	60	M	13	R	1	33	0/1	1/9	3/15	1	2	877	29	24	1	2
5	63	M	14	L	1	52	0/8	6/10	11/21	9	4	1331	45	36	1	4
6	66	M	10	L	1	41	0/5	2/7	6/20	9	3	1113	40	32	2	3
7	73	F	10	L	1	35	0/2	1/6	8/18	13	4	814	27	16	1	3
8	66	M	13	S	1	36	0/0	5/7	12/18	13	4	1916	30	24	2	3
9	71	M	8	S	4	20	2/6	0/3	2/10	4	1	891	13	7	1	1
Mean ± SE	64.4	-	9.4	-	(see key below)	37.0±0.9	-	-	-	8.1±0.6	-	-	31.3±3.0	24.8±2.9	1.2±0.2	-

UPDRS = Unified Parkinson's Disease Rating Scale; HY = Hoehn and Yahr stage.

† Side of onset: L = left, R = right, S = symmetrical onset.

* Tremor, rigidity, and bradykinesia were assessed on and off medication. Indication for surgery code: 1 = severe motor fluctuations with dyskinesias, 2 = resistant tremor, 3 = resistant tremor and motor fluctuations, 4 = resistant tremor and medication intolerance.

### Clinical assessments

Patients were assessed by an experienced neurologist (PB) using the Movement Disorder Society - Unified Parkinson's disease rating scale (MDS - UPDRS) parts III (motor signs of Parkinson's disease) and IV (motor complications, dyskinesias and motor fluctuations), as well as the Hoehn and Yahr (HY) staging system.

### Recording saccadic eye movements

Visually guided horizontal saccades were recorded using a miniaturised infra-red 1 kHz saccadometer, low-pass filtered at 250 Hz with 12 bit resolution [28]. Patients wore the device on their head, secured by an elastic strap and resting on the bridge of the nose; three built-in low-power lasers projected red 13 cd m^−2^ spots subtending some 0.1 degrees in a horizontal line in the midline at ±10 degrees [29]. Because the stimuli move exactly with the head, no head-restraint is necessary: sessions were therefore comfortable for the PD patient, especially for those suffering with severe dyskinesias.

In each trial the central fixation target was displayed for a random fore-period of 1.0–2.0 s. It then appeared to jump to one of the two peripheral positions, chosen at random, and remained illuminated until 200 ms after the end of the saccade. Participants were instructed to follow the target with their eyes as it moved. A single experimental run consisted of twenty calibration trials followed by 200 experimental trials, and lasted less than 10 minutes; aberrant records contaminated by excessive head movement and blinks were automatically removed by the software, which also determined the saccadic latency using a saccade-detection algorithm based on velocity and acceleration.

### Surgical procedure

**Figure 1:**
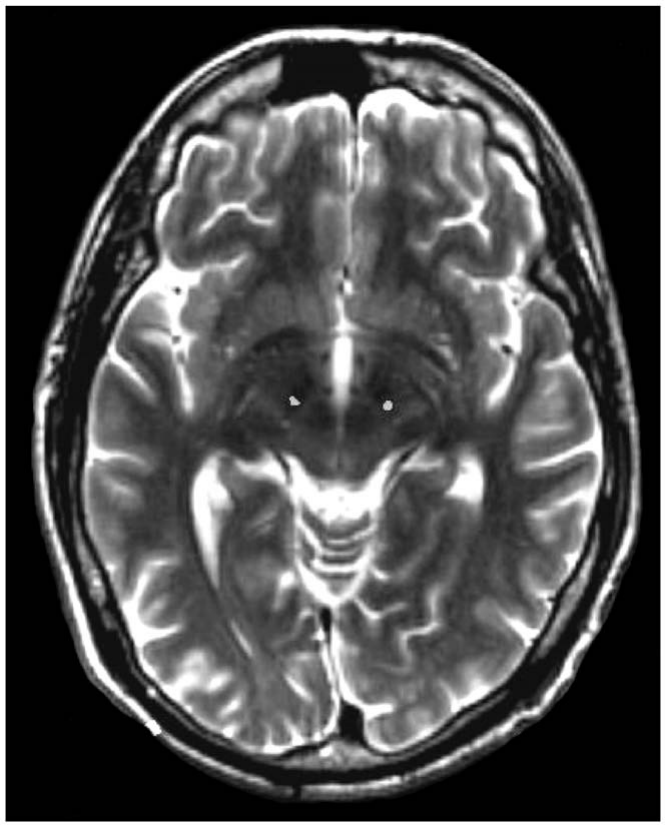
Confirmation of lead position: Postoperative thin slice CT windowed to show only the radio-dense leads (bright white spots) and fused to the preoperative T2 weighted MRI scan.

Prior to surgery a 3 Tesla magnetic resonance imaging (MRI) scan was obtained for each patient and checked to confirm good visualisation of the target anatomy. A Leksell stereotactic frame (Elekta, Sweden) was secured in position parallel to the AC-PC plane under local anaesthesia. A CT scan obtained with the frame in position was fused with the MRI scan using Framelink planning software (Medtronic, UK). Target co-ordinates were obtained for the subthalamic nucleus (STN), using direct anatomical targeting from the 3T MRI data set, and the stimulator lead (3389, Medtronic) advanced to target. The system was connected to a test stimulator set to deliver 60 µs pulses at 130 Hz. Neurological examination was performed as the stimulation current was slowly increased. Once satisfactory stimulation was confirmed the lead was secured in position and the process repeated on the opposite side. No electrode in this series was repositioned during surgery. A repeat CT scan was obtained and fused with the preoperative MRI scan to confirm the correct targeting. An example is shown in figure 1 where the leads are seen superimposed on the MRI image. The frame was then removed and under general anaesthesia the stimulator leads were tunnelled subcutaneously to a subclavicular pocket where they were connected to a Kinetra stimulator (Model 7428, Medtronic, UK).

The patient returned to the ward with the system remaining switched off. The patient was discharged on their original pharmacotherapy. Once they had recovered from surgery (three to four weeks postoperatively) the system was activated and titrated to clinical response in order to optimise the therapeutic effect.

### Protocol

All patients underwent saccadometry (a) preoperatively, (b) 24 hours postoperatively, (c) three to four weeks postoperatively, immediately prior to switching on the stimulator, and (d) following switch-on. General anaesthetic was administered only for the “tunneling” of the leads, for less than an hour. All patients were receiving their normal, uninterrupted, dopaminergic medication throughout this period, but because of the clinical priorities in this context, it was impossible to arrange for measurements to be made at precisely equivalent times after medication is taken. However, a previous study of combinations of medication and DBS [30] showed that medication per se had no significant effect of median latency, and if anything reduced the effects of DBS on latency. We did not measure latencies when the current was switched off again after ‘stimulation on’ because of the obvious ethical issues: this point had already been checked, and thoroughly confirmed, in previous studies [10], [11].

### Statistical analysis

The Kolmogorov-Smirnov two-sample statistic K-S2 [31], was used for comparing observed distributions, and one-sample statistic K-S1 for comparing observed with theoretical (LATER) distributions (http://www.cudos.ac.uk/later.html). Best-fit estimations of the LATER parameters were obtained by minimisation of the K-S1 statistic; no data set deviated significantly from the model. Since latency distributions are skewed, median latency (which is the reciprocal of the LATER μ parameter) was used as the preferred characteristic parameter. For comparing means of derived parameters in single subjects, the Student paired two-tailed *t*-test (exactly equivalent to repeated measures ANOVA) was used, having first confirmed compatibility with normal distributions of the parameter using the Shapiro-Wilk test. For comparisons between different timepoints, the paired two-tailed *t*-test was used for saccadic latencies, while the nonparametric sign test was used for clinical rating scores.

## Results

The preoperative and postoperative MDS-UPDRS parts III and IV scores and Hoehn and Yahr stages of all nine patients are shown in Table 1. The mean pre–operative UPDRS part III score was 37.0 (range 20–52) and the mean preoperative part IV score was 8.1 (range 1–13). One patient was HY stage 1, one stage 2, four stage 3, and three stage 4.

Even though they received no stimulation in the immediate postoperative period, all nine patients reported a symptomatic improvement as a result of the electrode placement, reflected in improvements in the UPDRS part III score. The mean UPDRS III at 24 hours postoperatively was 31.3 (range 13–45). The mean reduction in UPDRS III was 5.7 (range 1–11) and was significant with *p* = 0.002 (two sided sign test). This beneficial effect decayed away over the next three weeks.

Following switch-on of the stimulator there was substantial clinical improvement. UPDRS III fell to a mean of 24.8 (range 7–36), with a mean reduction of 12.2 (range 7–19), *p* = 0.002 (two sided sign test). UPDRS IV fell to a mean of 1.2 (range 0–2), mean reduction 6.9 (range 0–12), *p* = 0.004 (two sided sign test). Three patients' HY stage improved by one point, the other six being unchanged. Table 2 lists the actual coordinates of the electrode lead tips, obtained by fusing the postoperative CT with the original MRI scan, together with the stimulator parameters (voltage, frequency, pulse width, and contacts used), for each patient.

**Table 2:** Actual coordinates of lead tips, and stimulation parameters, for each patient.

Case	Side	Lead tip actual coordinates relative to mid AC-PC			Stimulation parameters			
		Anterior (y)	Lateral (x)	Vertical (z)	Contacts	Volts	Width	Freq
1	L	−1.8	−10.4	−4.0	1−,2−	3.5	60	130
	R	−2.7	11.6	−4.0	4−,5−	3.0	60	130
2	L	−2.7	−12.0	−4.5	0−,1−	2.0	60	130
	R	−2.7	11.6	−2.2	5+,7−	2.0	60	130
3	L	−2.4	−7.6	−4.5	1−	1.6	60	200
	R	−3.8	9.6	−4.5	7−	3.5	60	200
4	L	−5.5	−12.0	−4.4	2−	1.5	60	130
	R	−3.2	11.1	−4.4	5−	1.5	60	130
5	L	−2.4	−10.2	−4.9	2−,3−	1.8	60	130
	R	−1.8	14.9	−2.8	4−	2.0	60	130
6	L	−2.5	−8.9	−4.3	1−	2.0	60	130
	R	−3.7	9.0	−4.0	5−	2.0	60	130
7	L	−1.4	−12.3	−3.7	3+	1.4	60	130
	R	−1.4	12.2	−4.2	5+	1.5	60	130
8	L	−3.2	−12.0	−4.8	1−	2.5	60	130
	R	−4.0	11.7	−5.7	7−	2.5	60	130
9	L	−3.1	−14.6	−2.2	2−	2.0	60	130
	R	−4.9	10.3	−3.4	5−,6−	2.0	60	130

L = left lead, R = right lead; mid AC-PC = midpoint of line between anterior and posterior commisures.

**Figure 2:**
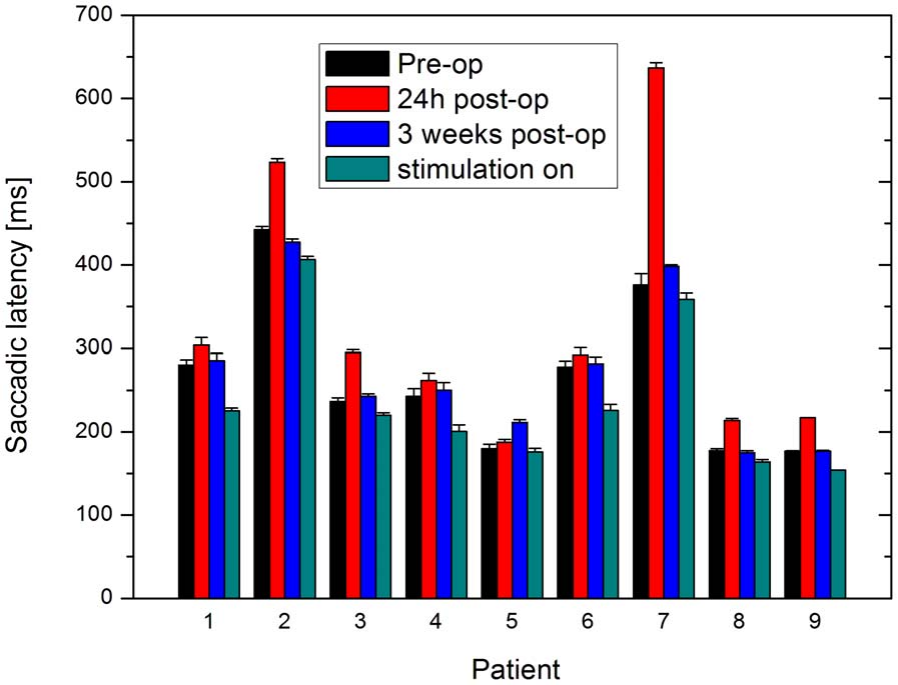
Median saccadic latency for each patient at the four time points. Black = pre-operative, red = 24 hrs post-operatively with stimulation off, blue = 3 weeks after operation with stimulation off, and green = immediately after switch on of stimulator.

**Figure 3:**
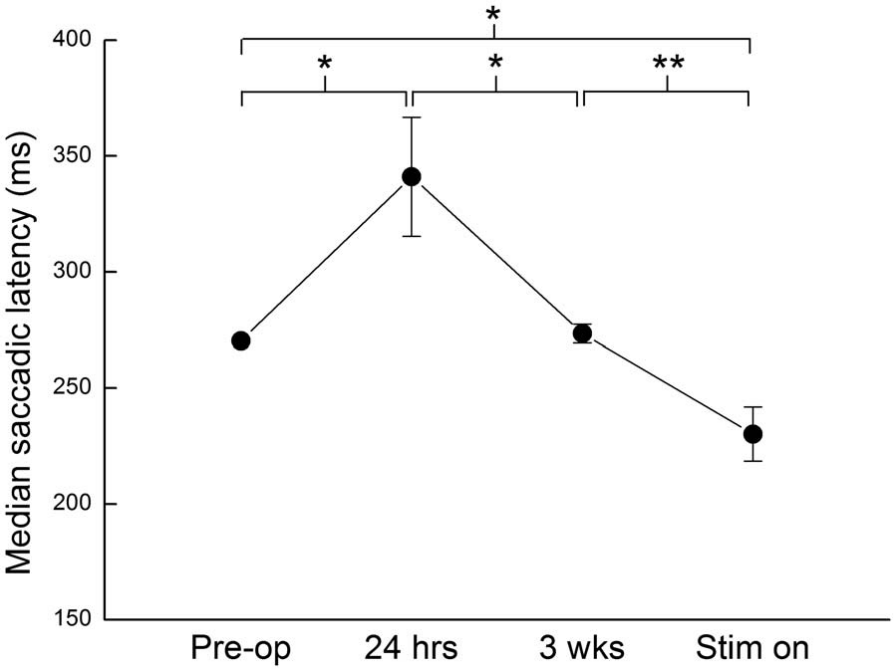
Median saccadic latency averaged over all nine patients, relative to the baseline, preoperative value; error bars show 1 S.E. Group comparisons between timepoints are illustrated at the top (* *p*<0.05, ** *p*<0.01).

The median saccadic latency for each patient at each time-point is shown in Figure 2, and the means of these latencies, averaged over all nine patients at each time-point and relative to the baseline pre-operative value, are shown in Figure 3; Table 3 summarises all the saccadic parameters.

**Table 3:** Saccadic LATER parameters (mean ± SE) and median latency, averaged over the nine patients.

	Pre –op	24 hrs	3 weeks	Stim On
μ (reciprocal median latency) (s^−1^)	4.05±0.39	3.41±0.40	3.93±0.37	4.75±0.42
σ (SD of main distribution) (s^−1^)	1.14±0.06	1.00±0.07	1.05±0.07	1.13±0.08
σ_E_ (SD of early distribution) (s^−1^)	4.70±0.43	4.19±0.38	4.46±0.34	3.49±0.63
Median Latency (ms)	270	341	276	231

As can be seen from figure 2, for every patient the median latency is higher 24 hours postoperatively than preoperatively. The mean rise in latency from baseline is 71 ms, and is significant with *p* = 0.02 (paired two-tailed *t*-test). Then – like the therapeutic benefit – this change in latency declines over the subsequent three weeks, in eight of the nine patients. At three weeks postoperatively, prior to switch-on, the mean latency is just 6 ms above baseline and the difference from baseline is no longer significant (*p* = 0.17, paired two-tailed *t*-test).

Following switch-on, in every case the latency decreases. Mean latency falls from 6 ms above baseline to 39 ms below. This is a highly significant change (*p* = 0.003, paired two-tailed *t*-test), and the difference from baseline after switch-on is also highly significant (*p* = 0.008, paired two-tailed *t*-test). Despite the wide variation in baseline latency, the pattern of changes over time was identical for all but one of the patients: a rise in latency postoperatively, a return to preoperative baseline values at three weeks, and then a fall in latency on switching on the stimulator. The exception was patient 5 (Figure 2), for whom latency rose slightly rather than falling over the post-operative period, before switch-on. Unlike ordinary measurements of mean latency with a much smaller number of trials, with saccadometry differences of latency of the sizes reported here can be statistically extremely significant. One of the main purposes of saccadometry is to gather enough data that not only do small differences become highly significant, but that the shape of the distribution itself can provide important information about the underlying neural processes. An example is one of the very first papers in this area, on the effects of sedative doses of anesthetic: [32] reporting SEs across six subjects of around 4 msec.

## Discussion

The shortening effect of STN DBS on prosaccadic latency has been previously documented [10], [11] and the data from this study reinforce these earlier results. That lead insertion alone can cause a transient improvement in symptoms, often termed ‘stunning’, is an equally well-established phenomenon, and can be seen clearly in our dataset as a small but significant fall in the UPDRS III score 24 hours after lead insertion, in the absence of stimulation. The efficacy and reliability of STN DBS in treating PD is very well established, and the larger fall in UPDRS III when the stimulator is switched on, as well as improvements in UPDRS IV and in some cases in HY stage, are entirely as expected. The novel finding in this paper is a very surprising one: that whereas DBS surgery and DBS stimulation both ameliorate the clinical signs of PD, the very large effects of DBS stimulation on saccadic latency are opposite in direction to what is seen as the result of surgery alone.

Certain anaesthetic drugs can affect saccadic latency and it is important to minimise the potential confounding effect they might have on postoperative saccadic data. In this group of patients, general anaesthesia was administered only for the tunnelling of the leads and insertion of the stimulator battery, for less than an hour, and the first postoperative measurements were made after a gap of 24 hours; it is very unlikely that significant residual effects of the anaesthetic would remain. The effects of small doses of volatile anaesthetics on saccadic latency distributions have already been quite thoroughly documented by Carpenter and colleagues [32], [33], [34].

The anatomical co-localisation of lesion and stimulation is an important consideration when trying to make sense of these findings. One problem is that the tissue disturbance and oedema caused during implantation, which one assumes to be the cause of the temporary lesioning effect, cannot be localised to a particular point such as the lead tip - it will affect the whole tract of the lead including the region around all four of its electrical contacts. Since none of the leads in this study were repositioned during surgery, the region of stimulation must be along this line, i.e. the region stimulated will co-localise with *part of* the area affected by oedema. In terms of the relative position of stimulation and oedema the variable quantity anatomically will be how long a section of lead there is deep to the area of stimulation. How important this is, we do not know. Table 2 lists the details of which contacts were used for stimulation in these patients (contacts 0,1,2,3 are on the left lead, and 4,5,6,7 on the right, with numbers 0 and 4 being closest to the lead tips). Use of higher numbered contacts means a greater distance between the lead tip and the contact used for stimulation; in this (admittedly small) group of patients we could discern no obvious pattern with respect to the magnitude of the lesion effect.

What might these findings imply about the underlying mechanism of STN DBS? At present we have really very little idea how deep brain stimulation works. Even the most fundamental question - does DBS essentially cause excitation or inhibition? – is currently controversial, with apparently conflicting results from different studies [12], [18], [19], [20], [21], [22]. Some of this confusion may be due to uncertainty about exactly what neurons DBS is acting on. Depending on the magnitude of the stimulating current, activation of neural elements may occur over distances of millimetres [19] and is therefore almost certain to stray outside the bounds of the nucleus, whose volume, in humans, is only some 240 mm^3^ [18]. As threshold current densities are lower for axons than for cell bodies, there is a greater likelihood of stimulating afferents to the STN (which are predominantly inhibitory) than the neurons of origin of the efferent excitatory pathways to substantia nigra pars reticulata (SNr) and globus pallidus internus (GPi). In addition, the neurons may respond to continual electrical stimulation with depolarisation block [19], [35], and there may be transmitter depletion [36], or the depressant effects of adenosine released from stimulated astrocytes [37]. Correspondingly, there is far from universal agreement about how STN DBS achieves its therapeutic effect [17], [38], [39], [40], [41]. An obvious interpretation of the beneficial changes in parkinsonism that occur as a result of electrode placement, and again after stimulator switch-on, is that both placement and stimulation are having similar effects on the STN, a functional impairment rather than enhancement. It can thus be viewed as analogous to subthalamotomy, which, although not often practised for fear of causing hemiballism, is therapeutic in PD [42], improving bradykinesia through reduced excitation of GPi and SNr.

Such an interpretation assumes that the lesions created by electrode penetration must reduce neural activity, but this is not necessarily true. Disruption of the afferent inhibitory projections to the STN could lead to an increase in its activity. Furthermore, the essentially negative effects of the associated neuronal destruction may also be accompanied by transient excitation because of increased leakage current in damaged dendrites of neurons whose somata remain intact. Given that dendritic fields, at least in rats [43] can extend over half the nucleus, this could be a functionally widespread effect. Furthermore, one must also take into account the fact that during insertion the electrode will be causing similar damage – again, possibly with a mixture of positive and negative effects – in distant structures with not very direct influence on the subthalamus, that may nevertheless have a functional relationship either with the initiation of saccades or with more general aspects of behaviour. An obvious possibility is frontal cortex, though in this study care was taken to ensure that the electrode track did not pass through either the frontal or supplementary eye fields. At deeper levels, the electrode will necessarily pass through the internal capsule and part of the thalamus, and may also influence the projection from the mediodorsal thalamus to the frontal eye fields [44] though passage through this region does not normally appear to evoke any obvious motor signs, it would clearly be desirable to undertake a systematic study of possible effects on oculomotor or more general motor function at the earlier stages of electrode advancement.

These afferent pathways, and the STN itself, are divided into zones corresponding to different kinds of input [45], and to a certain extent to the two major outputs of the STN, SNr (that in turn influences saccades through the superior colliculus) and GPi (that influences other kinds of movements through its thalamocortical projection) [46], [47]. This may perhaps provide an explanation for the otherwise puzzling difference that we have observed between saccadic latency and amelioration of PD symptoms. If the result of both electrode placement and stimulation is a complex balance between excitatory and inhibitory effects, then it is quite possible that this balance is different in each case as between oculomotor and more general effects. In addition, the STN sends a powerful glutamatergic projection to the substantia nigra pars reticulata [48], [49], [50] which generates disinhibition of the superior colliculus [51], part of a pathway descending from the cortex via the caudate nucleus and globus pallidus, that plays an important role in the initiation of saccades; these pathways are very likely anatomically distinct, and might again contribute to a different balance of excitation and inhibition in the two cases.

Clearly more detailed investigation is needed at the time of electrode insertion, including systematic exploration of different levels of stimulation that will produce different degrees of current spread. The STN is a mysterious structure, and exactly what these procedures are doing to it is equally mysterious. The fact that it has a rather central role specifically in saccadic control, and that saccadometry can generate relatively reliable quantitative data in a short period of time under clinical conditions, provides a valuable tool that may help elucidate how the function of the STN is modified in DBS.
